# Frequency‐ and Network‐Specific Changes in Functional Connectivity Reflect Pathophysiological Mechanisms across Parkinson's Disease Stages

**DOI:** 10.1002/ana.78262

**Published:** 2026-05-27

**Authors:** Matteo Conti, Valentina D'Onofrio, Luca Lorenzon, Laura Ludovica Grassi, Davide Mascioli, Valerio Ferrari, Clara Simonetta, Sofia Pavan, Francesca Di Giuliano, Mariangela Pierantozzi, Tommaso Schirinzi, Diego Centonze, Maurizio Corbetta, Angelo Antonini, Alessandro Stefani, Andrea Guerra

**Affiliations:** ^1^ Department of Systems Medicine University of Rome “Tor Vergata” Rome Italy; ^2^ Padova Neuroscience Center University of Padua Padua Italy; ^3^ Department of Neuroscience University of Padua Padua Italy; ^4^ Neurology Unit, IRCCS Neuromed Pozzilli Italy; ^5^ Neuroradiology Unit, Department of Biomedicine and Prevention University of Rome “Tor Vergata” Rome Italy; ^6^ Department of Neuroscience and Padova Neuroscience Center (PNC) University of Padova Padova Italy; ^7^ IRCCS San Camillo Hospital Venice Italy; ^8^ Parkinson Center Tor Vergata University Hospital Rome Italy

## Abstract

**Objective:**

Parkinson's disease (PD) is increasingly conceptualized as a disorder of large‐scale brain networks, yet whether and how frequency‐specific functional connectivity reorganizes across stages remains poorly understood. In this study, we used high‐density electroencephalography (EEG) to characterize cortico‐cortical functional connectivity across the clinical spectrum of PD.

**Methods:**

We performed high‐density EEG in a cross‐sectional cohort of 140 PD patients spanning early, intermediate, and advanced stages and 57 healthy controls. Cortico‐cortical functional connectivity was reconstructed in source space across multiple frequency bands and analyzed using network‐based statistics combined with machine‐learning models to identify stage‐dependent network alterations and evaluate their diagnostic and prognostic relevance.

**Results:**

We detected 3 distinct large‐scale networks showing divergent trajectories across disease stages. An α‐band network involving prefrontal and parieto‐temporal regions exhibited progressive hypoconnectivity and was associated with cognitive and axial impairment. A β‐band sensorimotor network showed progressive hyperconnectivity, paralleling bradykinesia severity. A high‐γ network demonstrated increased connectivity in early PD, followed by a progressive connectivity breakdown, and was inversely associated with motor complications. Multiband integration achieved near‐perfect discrimination between early PD and healthy controls and robust stratification across disease stages. Band‐specific networks also predicted clinical milestones of disease progression, with preserved α connectivity identifying patients at lower risk for cognitive and axial impairment, and stronger high‐*γ* connectivity indicating reduced vulnerability to motor complications.

**Interpretation:**

Together, these results identify frequency‐specific cortical networks as markers of disease stage and clinical vulnerability and support high‐density EEG connectivity as a scalable systems‐level biomarker for diagnosis, staging, and risk stratification in PD. ANN NEUROL 2026;100:319–333

Understanding how neurodegeneration disrupts large‐scale brain networks has become a central challenge in modern neuroscience.^1^ Rather than reflecting dysfunction of isolated regions, many neurological diseases are increasingly conceptualized as disorders of distributed networks,^2^ in which pathological processes propagate along structurally and functionally connected pathways.^3^, ^4^, ^5^ Parkinson's disease (PD) represents a paradigmatic model of network‐based neurodegeneration. Although classically defined by nigrostriatal dopaminergic degeneration, PD is now recognized as a multisystem disorder characterized by early and progressive involvement of widespread cortical and subcortical networks.^3^, ^6^, ^7^ Accumulating evidence suggests that pathological α‐synuclein aggregates propagate through anatomically and functionally connected brain regions, providing a biological substrate for large‐scale network disruption.^8^ Genetic factors, molecular co‐pathologies, and alterations of neurovascular integrity further shape network reorganization in PD, reinforcing the view of connectivity as an integrative systems‐level expression of disease biology rather than a mere consequence.^9^, ^10^, ^11^ Moreover, converging evidence from neuroimaging, neurophysiology, and fluid biomarkers in PD links the disruption of large‐scale functional networks to synaptic dysfunction, neurotransmitter system imbalance, and proteinopathy, thereby connecting network failure to both motor and non‐motor manifestations of the disease.^2^, ^3^, ^12^

Within this pathophysiological framework, neurophysiological studies have shown that cortico‐cortical functional connectivity (FC) alterations are early and consistent features of PD. Using high‐density electroencephalography EEG (HD‐EEG), we recently demonstrated that FC alterations in early‐stage PD are frequency‐ and network‐specific and map onto distinct neurobiological substrates. α‐Band hypoconnectivity in prefrontal networks was associated with cognitive impairment and gait disturbances, potentially reflecting cholinergic dysfunction, whereas β‐band hyperconnectivity in sensorimotor networks paralleled bradykinesia, which is consistent with dopaminergic circuit dysfunction.^7^, ^13^ Moreover, specific α‐band subnetwork alterations and β‐band network connectivity asymmetry differentiated early PD subtypes.^13^

Although these findings highlight HD‐EEG–based FC as a sensitive marker of early network disruption and subtype differentiation, current evidence remains largely confined to the initial stages of the disease. Particularly, it remains unclear whether abnormal patterns observed in early PD worsen over time, reorganize, or are accompanied by additional stage‐specific network signatures. Moreover, accumulating evidence suggests that cortical network reorganization may reflect not only progressive disease‐related dysfunction, but also compensatory processes aimed at preserving motor and cognitive functions.^14^, ^15^, ^16^ Whether compensatory and maladaptive patterns coexist with progressive network breakdown, and how they relate to symptoms and complications across disease stages, remains largely unknown. Clarifying these relationships would advance understanding of PD pathophysiology and establish the potential of HD‐EEG–based cortico‐cortical FC as a disease progression biomarker that is capable of capturing stage‐specific network dynamics and clinically meaningful milestones of disease evolution.

To address these gaps, we conducted a cross‐sectional study to assess FC alterations in a large, well‐characterized patient cohort spanning the full spectrum of disease severity across PD stages (early, intermediate, and advanced) using source‐level HD‐EEG and a standardized analytical pipeline. By integrating network‐based statistics (NBS) with machine learning (ML) approaches, we sought to characterize the trajectory of FC changes across disease stages, identify frequency‐specific networks most sensitive to disease progression, and determine their relationship with motor and non‐motor symptoms and disease‐related complications, as assessed using standardized clinical scales.

## Methods

### Study Population

Patients with PD were enrolled at the Movement Disorders clinics of the University of Padua (Padua, Italy) and Tor Vergata University Hospital (Rome, Italy) from January 2023 to June 2025. PD diagnosis was made according to the latest international criteria.^17^ Exclusion criteria were: (1) history of epilepsy or other neurological conditions that might have modified EEG; (2) major systemic (eg, cancer, organ failure, infections, and vascular accidents) and psychiatric disorders; and (3) cognitive impairment severe enough to prevent valid cooperation. Patients were classified as follows: early‐stage PD (ePD) if they had no motor fluctuations, for instance, Movement Disorders Society–Unified Parkinson's Disease Rating Scale part IV (MDS‐UPDRS IV) score = 0 and disease duration 3 years or less^7^; intermediate‐stage PD (iPD) if motor fluctuations were present, but the “5‐2‐1” criteria (≥5 doses of levodopa daily, ≥2 hours of daily OFF time, or ≥1 hour of daily troublesome dyskinesia^18^) for advanced‐stage PD (aPD) were not met; and aPD if they exhibited motor fluctuations and fulfilled the “5‐2‐1” criteria.^18^ An age‐ and sex‐matched group of healthy controls (HC) was also recruited, using the same exclusion criteria applied to patients. The present cohort was newly recruited for this study and was independent from those included in our previous research.^7^, ^13^ The study was approved by the local ethics committees of the participating institutions (protocols 16.21 and 16.17) and conformed to the principles of the Declaration of Helsinki. All participants signed an informed consent.

### Clinical Assessments

Demographic and clinical data were obtained for all participants on the same day as the EEG recording. Motor impairment in PD patients was assessed using the MDS‐UPDRS III and specific subscores (rigidity: 3.3; bradykinesia: 3.4–3.8, 3.14; tremor: 3.15–3.18; postural instability and gait disturbances [PIGD]: 3.9–3.13).^19^ The presence and severity of motor fluctuations were evaluated using the MDS‐UPDRS IV.^19^ Cognitive function was assessed using the Montreal Cognitive Assessment (MoCA).^20^ Non‐motor symptoms were evaluated using the Non‐Motor Symptoms Scale (NMSS).

To capture clinically meaningful milestones of PD progression, we defined 4 binary (yes/no) outcomes a priori: (1) motor fluctuations, (3) dyskinesia, (3) cognitive impairment, and (4) severe axial impairment. The presence of motor fluctuations (including wearing‐off, ON–OFF fluctuations, and delayed ON) and dyskinesia was derived from MDS‐UPDRS IV. Cognitive impairment was operationalized using MoCA‐based cut‐off (≤22) from a recent data‐driven model in PD^21^. Severe axial impairment was defined as the achievement of a 7‐point or more increase in the MDS‐UPDRS III PIGD subscore.^22^

### EEG Recording and Analysis

HD‐EEG recordings were acquired for 10 minutes at a sampling rate of 1,024 Hz and band‐pass filtered between 0.5 and 100Hz, using a 64‐channel EEG system. Electrodes were positioned according to the international 10–10 system, and impedance was maintained below 5kΩ. The first 5 minutes were acquired under eyes‐closed resting‐state condition, during which participants were instructed to keep their eyes closed while remaining awake and relaxed. Vigilance was monitored throughout acquisition, and all recordings were visually inspected by an experienced neurophysiologist before analysis. No participant showed EEG evidence of sleep onset during the selected resting‐state segments.^23^ The remaining 5 minutes were used to assess reactivity to eye opening and to perform routine activation procedures to exclude epileptiform activity. All PD patients underwent EEG recording in the OFF‐medication state, following overnight withdrawal of dopaminergic medication. An identical acquisition protocol was applied to HC.

Recordings were segmented into 30‐second epochs for visual inspection.^23^ The first epoch was discarded, and 6 consecutive low‐artifact epochs (totaling 180 seconds) were manually selected for analysis in both the PD and HC groups. Residual artifacts were removed using independent component analysis (ICA). Next, we proceeded with EEG source localization, based on the individual brain magnetic resonance imaging (MRI). FC was calculated in source space using weighted phase lag index (wPLI) in the θ (4–8Hz), α (8–13Hz), β (13–30Hz), low‐γ (30–50Hz), and high‐γ (50–100Hz) bands. Data analysis was performed using the Brainstorm toolbox,^24^ combined with custom‐written scripts for MATLAB R2025b (The MathWorks, Natick, MA). Details on the estimation of source activity and FC analysis are provided in the Supplementary Materials.

### Statistical Analysis of Demographic and Clinical Data

Because the Kolmogorov–Smirnov test indicated normality of the study variables, we used parametric statistics. Differences in demographic and clinical variables across the 4 groups (HC, ePD, iPD, and aPD) were assessed using Fisher's exact test for categorical data and 1‐way analysis of variance (ANOVA) for quantitative variables. Clinical measures specific to PD were compared across the 3 patient subgroups. Group differences in the prevalence of clinical milestones (motor fluctuations, dyskinesia, cognitive impairment, and severe axial impairment) were tested using Fisher's exact test or χ^2^ test when appropriate. All tests were 2‐tailed, with statistical significance set at *p* < 0.05. Effect sizes were also reported, using Cramér's V for Fisher's exact test (small = 0.1, medium = 0.3, large = 0.5), and partial η^2^ (ηp^2^) for ANOVA (small = 0.01, medium = 0.06, large = 0.14). Statistical analyses were conducted using IBM SPSS Statistics (IBM, Armonk, NY).

### Network‐Based Statistics for Group Comparisons

Group differences in θ, α, β, low‐γ, and high‐γ FC between PD patients and HCs were assessed using NBS^25^. This cluster‐level approach, widely applied in previous studies,^7^, ^13^, ^26^ offers higher sensitivity compared with conventional edge‐wise univariate testing and standard multiple‐comparison corrections.^25^ Further details on NBS analysis are provided in the Supplementary Materials.

Mean network connectivity (mNC) was defined as the average FC across all links within the altered network. Group differences in mNC among ePD, iPD, aPD, and HC were examined using analysis of covariance (ANCOVA), with age and sex included as covariates. Post hoc pairwise comparisons were conducted using the Tukey–Kramer procedure. Associations between mNC and clinical measures were assessed using partial Spearman's correlations. In the overall cohort, correlations were adjusted for age, sex, and group membership to account for disease stage effects. Additional analyses were performed separately within each group, adjusting for age and sex. Multiple comparisons were corrected using the false discovery rate (FDR). Furthermore, mNC was partitioned into interhemispheric and intrahemispheric components for each significant NBS‐derived network, and group differences were tested using the same ANCOVA framework. To assess whether one component contributed more strongly than the other to group discrimination, we also performed a bootstrap‐based paired comparison of their likelihood‐ratio improvements over a null multinomial model (5,000 iterations).

### ML‐Based Network Selection

Although control of the family‐wise error rate (FWER) is guaranteed irrespective of the NBS threshold, the choice of the primary threshold remains arbitrary. To address this issue, we combined NBS with ML procedures.^10^, ^11^

Specifically, we used a multiclass support vector machine (SVM) classifier including all diagnostic groups (HC, ePD, iPD, and aPD). Model performance was evaluated using nested cross‐validation, with leave‐one‐out cross‐validation (LOOCV) as the outer loop and Bayesian optimization in the inner loop for hyperparameter tuning.

**FIGURE 1:**
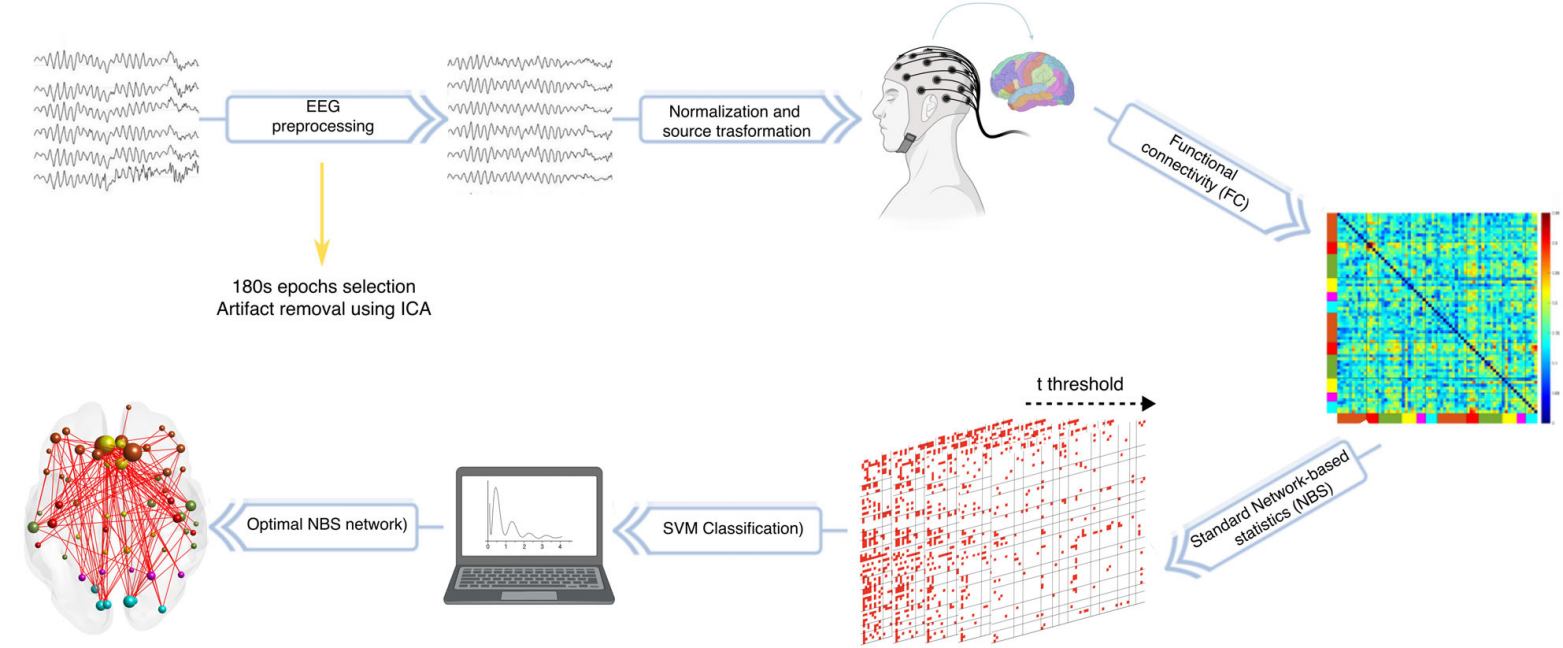
Overview of the electroencephalography (EEG) processing and network selection pipeline. Resting‐state EEG signals underwent preprocessing, including selection of artifact‐free 180‐second epochs and artifact removal using independent component analysis (ICA). Preprocessed data were normalized and projected into source space, and frequency‐specific functional connectivity (FC) matrices were computed at the source level. Standard network‐based statistics (NBS) was then applied across a range of t‐thresholds to identify candidate connectivity components. For each threshold, the corresponding NBS‐derived network was evaluated using a multiclass support vector machine (SVM) classifier. Classification performance across thresholds was used to identify the optimal NBS network, which was subsequently selected for downstream group‐level and clinical analyses. [Color figure can be viewed at www.annalsofneurology.org]

To identify the optimal NBS‐derived network, we examined a continuum of primary thresholds (from *F* = 2.0 in increments of 0.1). For each significant network identified across this range (*p* < 0.05), we applied the SVM classification pipeline described above. Cross‐validated classification accuracy was used as an empirical criterion to select the optimal NBS solution. The network achieving the highest accuracy was retained for subsequent analyses.^10^, ^11^, ^13^ The study pipeline is illustrated in Figure 1. Details on the estimation of ML‐based NBS selections are provided in the Supplementary Materials.

### Clinically Oriented Predictive Analyses of NBS‐Derived Networks

Using the NBS‐derived networks identified above, we conducted secondary clinically oriented analyses to evaluate: (1) binary discrimination between HC and ePD, (2) stage differentiation within the PD cohort (ePD vs iPD vs aPD), and (3) prediction of clinically relevant milestones of disease progression (motor fluctuations, dyskinesia, cognitive impairment, and severe axial impairment).

Binary and multiclass SVM classifiers were trained using network‐specific FC features. Model performance was evaluated using the same nested LOOCV and Bayesian hyperparameter optimization framework described above. Classification performance was quantified using confusion matrices and receiver operating characteristic (ROC) analyses. In particular, we quantified class‐specific performance using the model's confusion matrix. From this matrix, we derived multiple diagnostic metrics, including sensitivity (TPR), specificity (TNR), false‐positive rate (FPR), false‐negative rate (FNR), positive predictive value (PPV), FDR, negative predictive value (NPV), false omission rate (FOR), and accuracy. Moreover, we computed ROC curves for each class and their corresponding areas under the curve (AUCs).

To investigate whether complementary information across frequency bands improved predictive performance, we additionally constructed a multiband model combining all links belonging to the significant NBS networks identified across individual frequency bands and analyzed them using the same SVM‐based pipeline.

### Site‐Based External Validation Analysis

To further assess whether the classification findings generalized across independent recruitment sites, we conducted a complementary site‐based external validation. Specifically, the Padua cohort was used as the training and optimization dataset, whereas the Rome cohort was held out as an independent external validation dataset. SVM model training and optimization were performed exclusively within the Padua cohort. The trained models were then applied to the Rome cohort without further tuning. External validation performance was quantified using ROC analyses and confusion‐matrix‐derived metrics.

NBS and ML analyses were implemented in MATLAB 2025b using custom scripts together with the NBS and Classification Learner toolboxes. All statistical tests were 2‐tailed with significance set at *p* ≤ 0.05.

### A Priori Power Analysis

An a priori power analysis was conducted using G*Power 3.1^27^ to model the general linear model (ANCOVA) underlying the NBS analyses. We specified a 4‐level between‐subjects factor (groups: HC, ePD, iPD, and aPD) and 2 covariates (age and sex), assuming a medium effect size (*F* = 0.25, corresponding to partial η^2^ ≈ 0.06), α = 0.05, and power (1–β) = 0.80. This analysis indicated that a total sample of 179 participants would be required. Our final sample (n = 197) exceeded this target, ensuring adequate power to detect medium‐sized group effects at the network level.

## Results

A total of 140 PD patients and 57 age‐ and sex‐matched HC were included. Within the PD cohort, 60 participants were classified as ePD (42.8%), 40 as iPD (28.6%), and 40 as aPD (28.6%) (Table 1). The Padua cohort included 140 participants: 40 HC, 40 ePD, 30 iPD, and 30 aPD. The Rome cohort included 57 participants: 17 HC, 20 ePD, 10 iPD, and 10 aPD.

**TABLE 1:** Demographic and Clinical Characteristics of the Study Population.

Variable	ePD (n = 60)	iPD (n = 40)	aPD (n = 40)	HC (n = 57)	*p*	Effect size
Age (yr)	64.6 ± 9.4	63.7 ± 9.3	62.9 ± 6.5	66.0 ± 6.3	0.27	0.02
Sex (M/F)	40/20	24/16	25/15	32/25	0.70	0.09
Disease duration (yr)	1.9 ± 1.5	7.0 ± 3.4	12.9 ± 5.7	/	<**0.001**	0.61
MDS‐UPDRS III	22.6 ± 8.5	35.3 ± 10.2	46.1 ± 13.0	/	**<0.001**	0.48
Rigidity	4.4 ± 2.9	6.0 ± 7.9	7.9 ± 3.3	/	**<0.001**	0.18
Bradykinesia	10.2 ± 5.2	15.8 ± 5.4	46.1 ± 13.0	/	**<0.001**	0.41
Tremor	3.8 ± 3.5	5.1 ± 4.4	4.2 ± 6.0	/	0.36	0.02
PIGD	2.4 ± 1.8	4.3 ± 1.8	7.8 ± 3.7	/	**<0.001**	0.46
MDS‐UPDRS IV	0.0 ± 0.0	2.9 ± 3.1	8.6 ± 3.3	/	**<0.001**	0.48
LEDD	196.3 ± 222.3	598.7 ± 278.7	983.1 ± 461.9	/	**<0.001**	0.53
NMSS	30.0 ± 24.0	57.5 ± 40.7	70.7 ± 37.6	/	**<0.001**	0.22
MoCA	26.1 ± 2.7	24.2 ± 2.8	23.7 ± 2.9	26.3 ± 2.6	**<0.001**	0.12
Disease milestones (y/n)						
Motor fluctuations	0/60	20/20	35/5	/	**<0.001**	0.76
Dyskinesia	0/60	16/24	33/7	/	**<0.001**	0.72
Cognitive impairment	10/50	13/27	17/23	/	**0.02**	0.19
Severe axial impairment	3/57	7/33	24/16	/	**<0.001**	0.60

Data are expressed as mean ± standard deviation unless otherwise indicated. Statistically significant differences across groups are reported in bold. Effect sizes were reported for each comparison: Cramér's V for Fisher's exact test and partial η^2^ for ANOVAs.

aPD = advanced‐stage Parkinson's disease; ePD = early‐stage Parkinson's disease; F = female; HC = healthy controls; iPD = intermediate‐stage Parkinson's disease; LEDD = levodopa equivalent daily dose; M = male; MoCA = Montreal Cognitive Assessment; NMSS = Non‐Motor Symptoms Scale; y/n = yes/no; MDS‐UPDRS = Movement Disorder Society–Unified Parkinson's Disease Rating Scale; yr = year. Boldface indicates statistically significant (*p* < 0.05).

### FC Changes across Groups and Associations with Clinical Features

**FIGURE 2:**
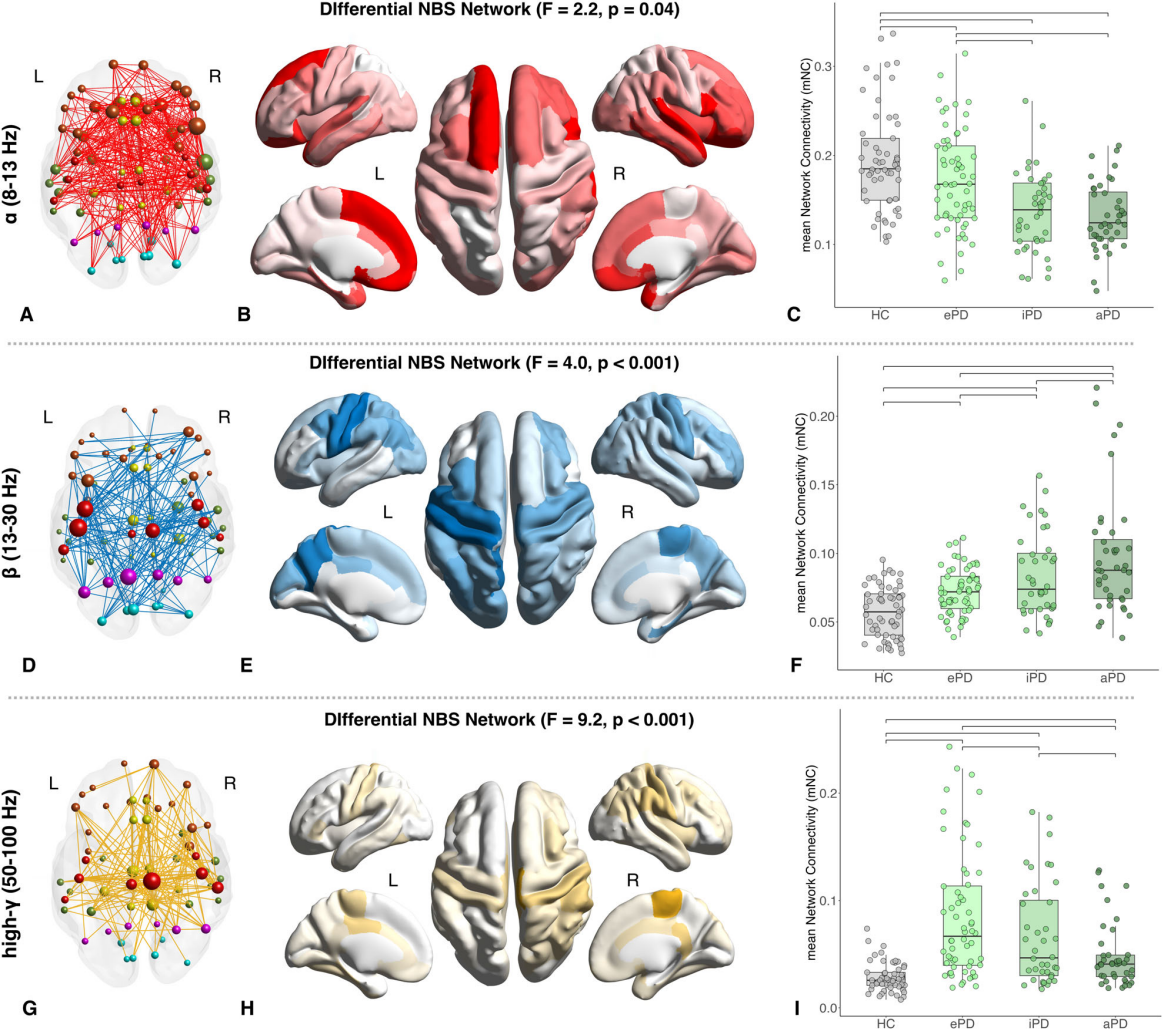
Differential NBS networks and frequency‐specific group effects across PD stages. (A–C) α‐Band (8–13 Hz) NBS network identified across groups (*F* = 2.2, *p* = 0.04). A shows the network topology with node size reflecting degree centrality; B illustrates its cortical projection; C displays the distribution of mean network connectivity (mNC) across diagnostic groups. (D–F) β‐band (13–30 Hz) NBS network (*F* = 4.0, *p* < 0.001) with similar visualization. (G–I) High‐γ band (50–100 Hz) NBS network (*F* = 9.2, *p* < 0.001). Boxplots in panels C, F, and I show groupwise differences in mNC values. Asterisks indicate significant pairwise comparisons. aPD = advanced‐stage PD; ePD = early‐stage PD; HC = healthy controls; iPD = intermediate‐stage PD; mNC = mean network connectivity; NBS = network‐based statistics; PD = Parkinson's disease. [Color figure can be viewed at www.annalsofneurology.org]

In the α frequency band, NBS identified a network that discriminated among groups (*F* = 2.2, *p* = 0.04, ηp^2^ = 0.04). This network comprised 68 nodes and 382 links, with a slight right hemisphere predominance (58.6%). Regions of interest (ROIs) with the highest degree were located in prefrontal (right ventrolateral prefrontal cortex, bilateral orbitofrontal cortices, bilateral supplementary motor areas, and dorsomedial prefrontal cortices), temporal (bilateral temporal poles, and right superior and inferior temporal gyri), and limbic (bilateral anterior cingulate cortices) regions. The most represented connections were fronto‐temporal (24.6%), intra‐frontal (16.0%), and fronto‐limbic (9.2%). The mNC differed significantly across groups (*F* = 9.02, *p* < 0.001), with higher values in HC than all PD subgroups (HC vs ePD, *p* = 0.04; HC vs iPD, *p* < 0.001; HC vs aPD, *p* < 0.001). ePD showed higher mNC than iPD (*p* = 0.02) and aPD (*p* = 0.004), whereas iPD and aPD did not differ (*p* = 0.97) (Fig 2A–C).

A second network distinguishing groups emerged in the β band (*F* = 4.0, *p* < 0.001, ηp^2^ = 0.16). This network included 66 nodes and 180 links, with a balanced distribution between hemispheres (right: 51.5%). High‐degree ROIs were located in the sensorimotor cortices (bilateral precentral and postcentral and bilateral supramarginal areas), parietal areas (bilateral precuneus and superior parietal lobule and left inferior parietal lobule), and premotor cortices. The most represented connections were fronto‐sensorimotor (11.1%), intra‐sensorimotor (6.7%), and intra‐frontal (6.1%). Group differences in β‐band mNC were robust (*F* = 19.1, *p* < 0.001), with lower values in HC than in all PD subgroups (HC vs ePD, *p* = 0.01; HC vs iPD, *p* < 0.001; HC vs aPD, *p* < 0.001) and progressively higher values across disease stages (iPD > ePD, *p* = 0.03; aPD > ePD, *p* < 0.001; aPD > iPD, *p* = 0.04) (see Fig 2D–F).

A third differential network was identified in the high‐γ band (*F* = 9.2, *p* < 0.001, ηp^2^ = 0.09). This network comprised 60 nodes and 161 links, with a bilateral distribution (right: 51.7%). High‐degree ROIs involved the sensorimotor cortex (bilateral precentral, postcentral, and paracentral ROIs), limbic areas (bilateral anterior and posterior cingulate), and prefrontal regions (right frontal pole and middle frontal gyrus). The most represented connections were fronto‐sensorimotor (13.7%), limbic‐sensorimotor (13.7%), temporo‐sensorimotor (13.0%), and limbic‐frontal (6.8%). High‐γ mNC varied significantly among groups (*F* = 25.3, *p* < 0.001). HC exhibited markedly lower values than all PD subgroups (HC vs ePD, *p* < 0.001; HC vs iPD, *p* < 0.001; HC vs aPD, *p* = 0.04). Within the PD cohort, high‐γ mNC showed a progressive reduction from early to advanced stages (ePD > iPD, *p* = 0.03; ePD > aPD, *p* < 0.001; iPD > aPD, *p* = 0.04) (see Fig 2F–H).

For all identified differential networks, separate analyses of intra‐ and interhemispheric mNC showed that both connectivity components followed the same stage‐dependent trajectory observed for global mNC (*α* network interhemispheric: *F* = 9.38, *p* < 0.001; α network intrahemispheric: *F* = 8.16, *p* < 0.001; β network interhemispheric: *F* = 14.77, *p* < 0.001; *β* network intrahemispheric: *F* = 19.36, *p* < 0.001; high‐γ network interhemispheric: *F* = 24.96, *p* < 0.001; high‐γ network intrahemispheric: *F* = 24.52, *p* < 0.001). Moreover, direct comparisons of the magnitude of group‐related changes revealed no differences between intra‐ and interhemispheric components (α network: *p* = 0.29, β network: *p* = 0.57, high‐γ network: *p* = 0.60), suggesting that the observed stage‐dependent alterations were not selectively driven by a single component.

**FIGURE 3:**
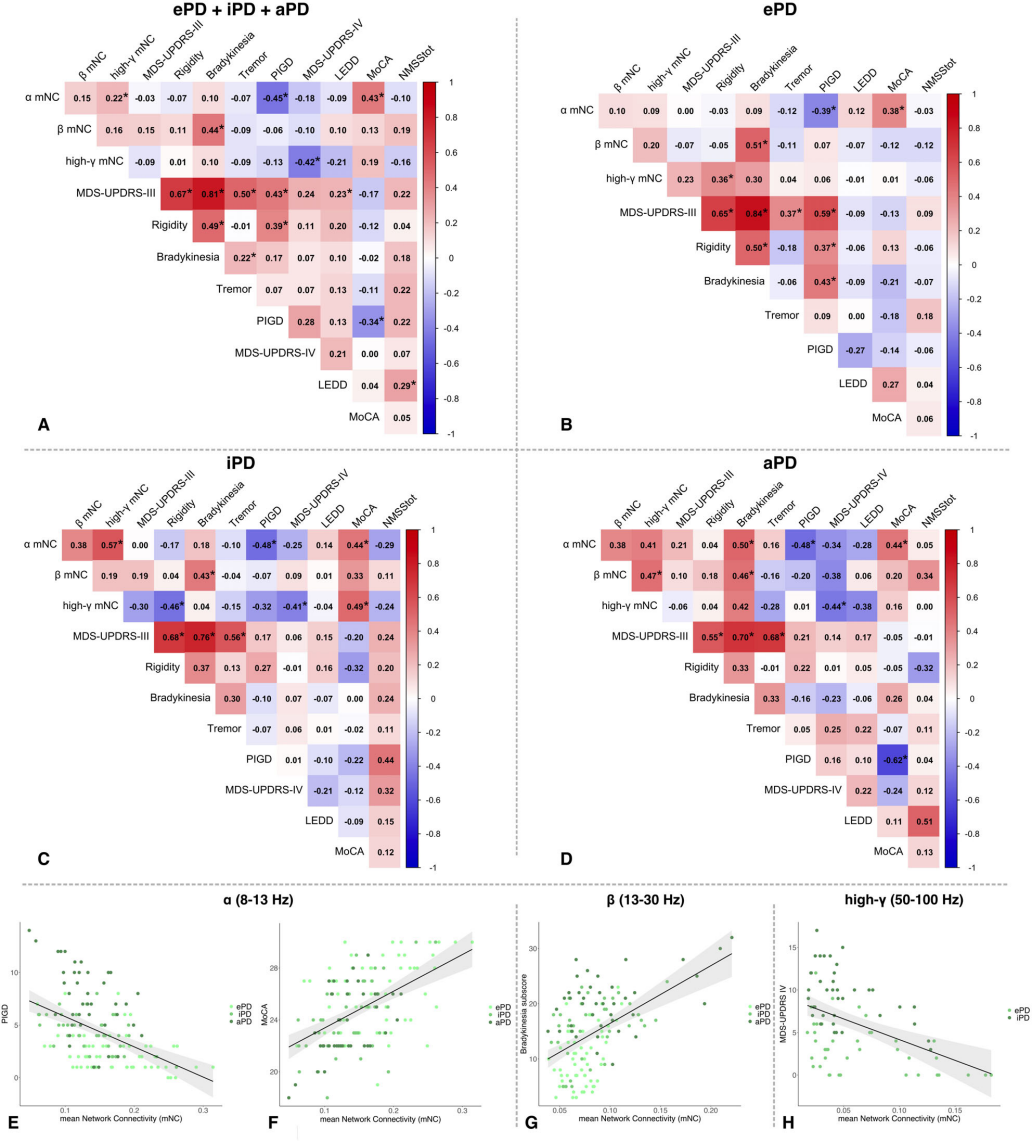
Correlation between frequency‐specific mNC and clinical features across PD stages. Correlation matrix showing associations between mNC in the α‐, β‐, and high‐γ‐band NBS networks and clinical variables in the overall PD cohort (ePD + iPD + aPD) (A). Stage‐specific correlation matrices for ePD, iPD, and aPD subgroups (B–D). Scatterplots show representative associations between mNC and selected clinical measures across the entire PD cohort (E–H). All correlations were computed using partial Spearman's rank correlation, adjusting for age, sex, and disease duration. *P*‐values were corrected for multiple comparisons using the false discovery rate (FDR) method. aPD = advanced‐stage PD; ePD = early‐stage PD; iPD = intermediate‐stage PD; LEDD = levodopa equivalent daily dose; MDS‐UPDRS = Movement Disorder Society Unified Parkinson's Disease Rating Scale; mNC = mean network connectivity; MoCA = Montreal Cognitive Assessment; NBS = network‐based statistics; NMSStot = total Non‐Motor Symptoms Scale score; PD = Parkinson's disease; PIGD = Postural Instability and Gait Disorder subscore. [Color figure can be viewed at www.annalsofneurology.org]

Across the PD cohort, frequency‐specific associations emerged between mNC and clinical variables. Lower α band mNC was linked to greater severity of PIGD (r = −0.45, *p* < 0.001) and lower MoCA scores (r = 0.43, *p* < 0.001). In the β band network, mNC correlated positively with bradykinesia severity (r = 0.44, *p* < 0.001). In contrast, high‐γ band mNC was negatively associated with the MDS‐UPDRS IV scores (r = −0.42, *p* < 0.001), indicating that higher high‐γ connectivity was linked to milder motor complications. These clinical–neurophysiological associations were confirmed within iPD and aPD subgroups. Of note, correlations with MDS‐UPDRS IV were not assessed in ePD as motor complications were absent in this cohort (Fig 3). No correlation was found between levodopa equivalent daily dose (LEDD) and α‐, β‐, or high‐γ‐band mNC values, either in the overall PD cohort or in stage‐specific analyses after multiple‐comparison correction.

### Classification Performance for Group Differentiation

**FIGURE 4:**
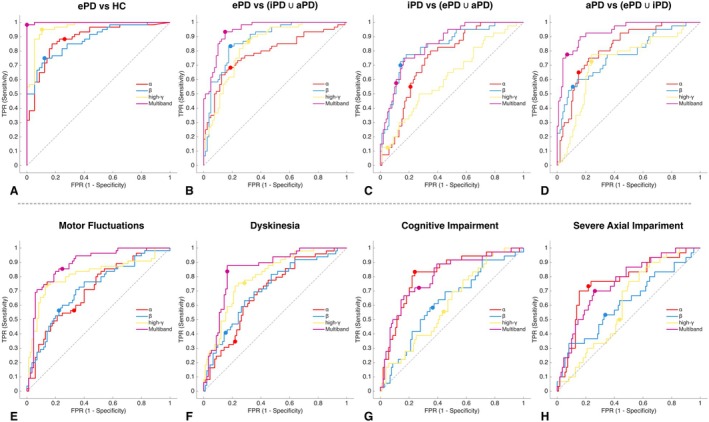
Receiver operating characteristic (ROC) curves for classification tasks based on NBS‐derived EEG functional networks. A–D show ROC curves for group‐level classification tasks, including differentiation between ePD and HC (A), and 1‐vs‐rest classification of ePD (B), iPD (C), and aPD (D). E–H show ROC curves for predicting clinical milestones (yes vs no), including motor fluctuations (E), dyskinesia (F), cognitive impairment (G), and severe axial impairment (H). For each panel, classification performance is reported for polynomial‐kernel SVM models trained on frequency‐specific NBS networks in the α (red), β (blue), and high‐γ (yellow) bands, as well as for a multiband model integrating all frequency‐specific predictors (magenta). aPD = advanced‐stage PD; ePD = early‐stage PD; iPD = intermediate‐stage PD; NBS = network‐based statistics; PD = Parkinson's disease; SVM = support vector machine. [Color figure can be viewed at www.annalsofneurology.org]

Driven by clinical translation purposes, we first evaluated the ability of NBS‐derived EEG functional networks to differentiate ePD patients from HCs (Fig 4A). The α‐ and β‐band networks showed good discriminative performance, with balanced sensitivity and specificity, and good accuracy. The high‐γ network achieved superior performance, yielding very high sensitivity and specificity with excellent accuracy in group discrimination. Notably, integrating frequency‐specific networks into a multiband model resulted in a near‐perfect discrimination between ePD and HC (AUC = 0.99), with excellent sensitivity (0.98), specificity (1.00), positive predictive value (1.00), NPV (0.98), and accuracy (0.99).

We, then, assessed the ability of NBS‐derived network features to discriminate among PD stages (see Fig 4B–D). When considered individually, all frequency bands provided good discriminative performance for ePD identification, with moderate‐to‐good classification accuracy, good sensitivity (high‐γ), specificity (α), or both (β). The multiband model again achieved the best overall performance, yielding an AUC of 0.94 with excellent sensitivity (0.93), good specificity (0.85), and accuracy (0.89). Classification of iPD proved more challenging across models, with the β‐band network showing the best single‐frequency performance (AUC = 0.83, specificity = 0.86, sensitivity = 0.70, accuracy = 0.81), and no improvement using the multiband integration model. For aPD identification, discriminative performance increased across most frequency bands, demonstrating fair‐to‐good classification ability and good specificity and accuracy. The multiband model again outperformed single‐band approaches, achieving an AUC of 0.93, excellent specificity (0.93), and good‐to‐excellent accuracy (0.89), supporting its robustness in detecting advanced disease stages.

Detailed classification metrics are reported in Tables 2 and 3.

**TABLE 2:** Classification Performance of Frequency‐Specific and Multiband NBS‐Derived Networks in Differentiating ePD Patients from HCs.

Metric	ePD vs HC			
	α	β	h‐γ	Multi
AUC	**0.89**	**0.88**	**0.96**	**1.00**
Sensitivity (TPR)	**0.88**	0.75	**0.95**	**0.98**
Specificity (TNR)	0.75	**0.88**	**0.89**	**1.00**
FNR (type II error)	0.12	0.25	0.05	0.02
FPR (type I error)	0.26	0.12	0.11	0.00
PPV	0.78	**0.87**	**0.90**	**1.00**
FDR	0.22	0.13	0.10	0.00
NPV	**0.86**	0.77	**0.94**	**0.98**
FOR	0.14	0.23	0.06	0.02
Accuracy	**0.81**	**0.81**	**0.92**	**0.99**

The table reports performance metrics from polynomial‐kernel SVM models trained on α‐, β‐, and high‐γ‐band NBS networks, as well as on a multiband model integrating all frequency‐specific predictors.

Accuracy = overall correct classification rate; AUC = area under the curve; ePD = early‐stage Parkinson's disease; HC = healthy controls; FDR = false discovery rate; FNR = false negative rate (or type II error); FOR = false omission rate; FPR = false positive rate (or type I error); NBS = network‐based statistics; NPV = negative predictive value; PPV = positive predictive value; SVM = support vector machine; TNR = true negative rate (or specificity); TPR = true positive rate (or sensitivity). Bold values indicate performance metrics ≥0.80. Error‐rate metrics, for which lower values indicate better performance, are not bolded.

**TABLE 3:** Classification Performance Metrics (1‐vs‐Rest) for Each PD Group (ePD, iPD, and aPD) across Frequency‐Specific NBS Networks and the Combined Multiband Model.

Metric	ePD vs (iPD ∪ aPD)				iPD vs (ePD ∪ aPD)				aPD vs (ePD ∪ iPD)			
	α	β	h‐γ	Multi	α	β	h‐γ	Multi	α	β	h‐γ	Multi
AUC	0.79	**0.88**	**0.84**	**0.94**	0.75	**0.83**	0.62	**0.85**	**0.82**	0.77	0.76	**0.93**
Sensitivity (TPR)	0.68	**0.83**	**0.87**	**0.93**	0.55	0.70	0.13	0.58	0.65	0.55	0.73	0.78
Specificity (TNR)	**0.81**	**0.81**	0.69	**0.85**	0.79	**0.86**	**0.95**	**0.89**	**0.85**	**0.89**	0.76	**0.93**
FNR (type II error)	0.32	0.17	0.13	0.07	0.45	0.30	0.88	0.43	0.35	0.45	0.28	0.23
FPR (type I error)	0.19	0.19	0.31	0.15	0.21	0.14	0.05	0.11	0.15	0.11	0.24	0.07
PPV	0.73	0.77	0.68	**0.82**	0.51	0.67	0.50	0.68	0.63	0.67	0.55	**0.82**
FDR	0.27	0.23	0.32	0.18	0.49	0.33	0.50	0.32	0.37	0.33	0.45	0.18
NPV	0.77	**0.87**	**0.87**	**0.94**	**0.81**	**0.88**	0.73	**0.84**	**0.86**	**0.83**	**0.87**	**0.91**
FOR	0.23	0.13	0.13	0.06	0.19	0.12	0.27	0.16	0.14	0.17	0.13	0.09
Accuracy	0.76	**0.82**	0.76	**0.89**	0.72	**0.81**	0.71	**0.80**	0.79	0.79	0.75	**0.89**

The table reports performance metrics from polynomial‐kernel SVM models trained on α‐, β‐, and high‐γ‐band NBS networks, as well as on a multiband model integrating all frequency‐specific predictors.

Accuracy = overall correct classification rate; aPD = advanced‐stage Parkinson's disease; AUC = area under the curve; ePD = early‐stage Parkinson's disease; FDR = false discovery rate; FNR = false negative rate (or type II error); FOR = false omission rate; FPR = false positive rate (or type I error); iPD = intermediate‐stage Parkinson's disease; NBS = network‐based statistics; NPV = negative predictive value; PPV = positive predictive value; SVM = support vector machine; TNR = true negative rate (or specificity); TPR = true positive rate (or sensitivity). Bold values indicate performance metrics ≥0.80. Error‐rate metrics, for which lower values indicate better performance, are not bolded.

### Site‐Based External Validation Analysis

For ePD versus HC discrimination, external validation confirmed good‐to‐excellent performance of the frequency‐specific models. The α‐band model achieved an AUC of 0.88 and accuracy of 0.73; the β‐band model achieved an AUC of 0.85 and accuracy of 0.76; and the high‐γ model achieved an AUC of 0.95 and accuracy of 0.89. The multiband model achieved perfect performance in external validation (AUC = 1.00, sensitivity = 1.00, specificity = 1.00, accuracy = 1.00) (Fig S1A). Detailed classification metrics are reported in Table S1.

For PD stage classification, the multiband model showed strong performance in external validation. For ePD versus iPD/aPD, it achieved an AUC of 0.94, sensitivity of 0.90, specificity of 0.79, and accuracy of 0.85. For iPD versus ePD/aPD, classification remained more challenging, but still showed good overall discrimination (AUC = 0.89, sensitivity = 0.56, specificity = 0.93, accuracy = 0.85). For aPD versus ePD/iPD, external validation performance was excellent (AUC = 0.99, sensitivity = 0.90, specificity = 0.97, accuracy = 0.95) (Fig S1B–D). Detailed classification metrics are reported in Table S2.

### Predictive Performance for Clinical Milestones of Disease Progression

Band‐specific EEG network models showed a frequency‐dependent improvement in discriminative performance for motor fluctuations, with accuracy increasing from lower to higher frequencies (see Fig 4E). The high‐γ network provided the best single‐band performance (AUC = 0.81), with good specificity (0.86), NPV (0.84), accuracy (0.81), and acceptable sensitivity (0.75). Multiband integration model further improved performance (AUC = 0.88), increasing sensitivity (0.85) and NPV (0.89), although decreasing specificity (0.75). A similar frequency‐dependent pattern was observed for dyskinesia prediction (see Fig 4F). The high‐γ network again showed the best single‐band performance (AUC = 0.80, sensitivity = 0.76, specificity = 0.71, NPV = 0.84, and accuracy = 0.73). The multiband model improved overall classification, reaching an AUC of 0.85 and achieving balanced sensitivity (0.84) and specificity (0.84) with excellent NPV (0.90).

In contrast, prediction of cognitive impairment and severe axial impairment was dominated by lower‐frequency network alterations (see Fig 4G,H). The α‐band network demonstrated the best overall performance, with good discriminative ability (AUC = 0.81 for cognitive impairment; AUC = 0.76 for axial impairment), acceptable specificity (0.76 and 0.78, respectively), and good accuracy (0.78 and 0.77, respectively). The sensitivity was good for cognitive impairment (0.83) and acceptable for axial impairment (0.73). Notably, the *α*‐band network demonstrated excellent NPV for both cognitive impairment (0.93) and severe axial impairment (0.91). The multiband model did not substantially outperform the α‐band network for these outcomes, suggesting that lower‐frequency network alterations contribute predominantly to cognitive decline and axial disturbances in PD. Table 4 shows a comprehensive summary of classification metrics.

**TABLE 4:** Classification Performance Metrics for Clinical Milestones across Frequency‐Specific NBS Networks and the Combined Multiband Model.

Metric	Motor fluctuations (yes vs no)				Dyskinesia (yes vs no)				Cognitive impairment (yes vs no)				Severe axial impairment (yes vs no)			
	α	β	h‐γ	Multi	α	β	h‐γ	Multi	α	β	h‐γ	Multi	α	β	h‐γ	Multi
AUC	0.71	0.71	**0.81**	**0.88**	0.69	0.71	**0.80**	**0.85**	**0.81**	0.62	0.61	**0.80**	0.76	0.62	0.61	0.76
Sensitivity (TPR)	0.56	0.56	0.75	**0.85**	0.35	0.41	0.76	**0.84**	**0.83**	0.58	0.56	0.72	0.73	0.53	0.50	0.70
Specificity (TNR)	0.67	0.78	**0.86**	0.75	0.78	0.85	0.71	**0.84**	0.76	0.63	0.56	0.73	0.78	0.66	0.56	0.74
FNR (type II error)	0.44	0.44	0.25	0.15	0.65	0.59	0.24	0.16	0.17	0.42	0.44	0.28	0.27	0.47	0.50	0.30
FPR (type I error)	0.33	0.22	0.14	0.25	0.22	0.15	0.29	0.16	0.24	0.37	0.44	0.27	0.22	0.34	0.44	0.26
PPV	0.53	0.62	0.77	0.69	0.46	0.59	0.59	0.73	0.55	0.36	0.30	0.48	0.48	0.30	0.24	0.42
FDR	0.47	0.38	0.23	0.31	0.54	0.41	0.41	0.27	0.45	0.64	0.70	0.52	0.52	0.70	0.76	0.58
NPV	0.70	0.73	**0.84**	**0.89**	0.69	0.73	**0.84**	**0.90**	**0.93**	**0.81**	0.78	**0.88**	**0.91**	**0.84**	**0.81**	**0.90**
FOR	0.30	0.27	0.16	0.11	0.31	0.27	0.16	0.10	0.07	0.19	0.22	0.12	0.09	0.16	0.19	0.10
Accuracy	0.63	0.69	**0.81**	0.79	0.63	0.69	0.73	**0.84**	0.78	0.62	0.56	0.73	0.77	0.64	0.55	0.73

The table reports performance metrics from polynomial‐kernel SVM models trained to discriminate the presence of clinically relevant disease milestones, using α‐, β‐, and high‐γ‐band NBS networks, as well as a multiband model integrating all frequency‐specific predictors.

Accuracy = overall correct classification rate; AUC = area under the curve; FDR = false discovery rate; FOR = false omission rate; FNR = false negative rate (or type II error); FPR = false positive rate (or type I error); NBS = network‐based statistics; NPV = negative predictive value; PPV = positive predictive value; SVM = support vector machine; TNR = true negative rate (or specificity); TPR = true positive rate (or sensitivity). Bold values indicate performance metrics ≥0.80. Error‐rate metrics, for which lower values indicate better performance, are not bolded.

## Discussion

In this study, we identified large‐scale frequency‐specific cortico‐cortical networks exhibiting distinct trajectories across PD stages. An α‐band network, involving predominantly prefrontal, sensorimotor, and parieto‐temporal regions, showed progressive hypoconnectivity, which correlated with PIGD severity and cognitive performance. A β‐band network encompassing sensorimotor, prefrontal, and limbic areas exhibited progressive hyperconnectivity and was associated with bradykinesia severity. Moreover, a high‐γ network showed higher connectivity in ePD than HC, followed by a progressive decline across disease stages and was negatively associated with motor complications severity. A multiband model integrating all frequency‐specific alterations exhibited excellent performance for PD diagnosis and disease stage identification, while α‐ and high‐γ network abnormalities had a high NPV for key clinical milestones of disease progression. Together, these findings reveal frequency‐ and network‐specific patterns of altered cortico‐cortical connectivity in PD, whose trajectories vary across disease stages and map onto distinct clinical manifestations.

Consistent with prior literature,^7^, ^10^, ^11^, ^13^ we confirmed reduced α‐band connectivity within a predominantly prefrontal and parieto‐temporal network and increased β‐band connectivity within a sensorimotor network in PD. α‐Band alterations are thought to reflect dysfunction of cholinergic‐dependent cortico‐cortical communication,^28^, ^29^, ^30^, ^31^ whereas β‐band hyperconnectivity has been interpreted as a marker of dopaminergic motor network dysfunction.^32^, ^33^, ^34^ The novel contribution of the present study is the demonstration that these well‐established band‐specific network alterations evolve along distinct stage‐dependent trajectories rather than representing static disease features. Specifically, α‐band connectivity declined progressively from early to advanced PD, suggesting cumulative disruption of large‐scale cortical integration as the disease progresses. This stage‐dependent reduction was closely associated with worsening cognitive performance and axial motor impairment, supporting the view that progressive α‐band disconnection reflects the increasing impact of non‐dopaminergic, particularly cholinergic, dysfunction. Rather than representing a static trait of early PD, α‐band hypoconnectivity appears to track disease progression, possibly reflecting the gradual loss of α‐band cortico‐cortical mechanisms supporting cognition and gait stability. Conversely, β‐band connectivity increased across disease stages, indicating progressive reinforcement of a hypersynchronous motor network. The presence of β‐band hyperconnectivity in early PD, followed by further increase with disease progression, suggests that abnormal β synchronization is both an early pathophysiological feature and a marker of ongoing network maladaptation. Moreover, the strong and consistent association between β‐band connectivity and bradykinesia severity across all disease stages further supports β hypersynchronization as a quantitative marker of motor disease progression rather than a simple early‐state abnormality.

The assessment of a large patient cohort across different disease stages enabled the identification of a novel pattern of FC abnormality in PD. We detected a high‐γ network involving sensorimotor, prefrontal, and limbic regions that demonstrated increased connectivity in early PD compared with controls, but progressively declined with advancing disease. This trajectory differs from both the α‐ and β‐band alterations, suggesting a distinct pathophysiological mechanism. In the motor system, cortical high‐γ oscillations increase during movement and are generally considered pro‐kinetic, emerging alongside suppression of pathological β synchrony within the cortico‐basal loop.^35^, ^36^ Dopaminergic therapy reduces exaggerated β activity while concomitantly increasing high‐γ activity, supporting the view that motor improvement reflects a shift toward more flexible high‐frequency dynamics, rather than a simple frequency‐specific spectral power change.^37^ Experimental evidence also suggests that high‐γ activity may facilitate plasticity processes in the primary motor cortex in PD.^38^, ^39^, ^40^ Within this framework, our findings suggest that increased synchronization in the high‐γ network may represent a compensatory upregulation that is progressively lost as neurodegeneration advances. This interpretation is supported by the negative association between high‐γ connectivity and motor complications severity: patients with stronger high‐γ synchronization exhibited fewer and less severe motor fluctuations, indicating that coordinated high‐γ activity may confer resilience against late‐stage motor system instability. At first glance, these findings may appear to contrast with reports linking excessively high‐γ power in the primary motor cortex to levodopa‐induced dyskinesia.^41^, ^42^ However, these phenomena likely reflect different aspects of γ‐band dynamics. Dyskinesia‐related γ activity is typically high‐amplitude, spectrally narrow, and locally generated, representing an aberrant dopamine‐dependent resonant state rather than an enhancement of physiological pro‐kinetic signaling. In contrast, the high‐γ FC measure captured here reflects network‐level cortico‐cortical synchronization indexing coherent information flow across distributed motor, prefrontal, and limbic networks, which is more consistent with adaptive motor processing. Although these findings point to a compensatory network mechanism, longitudinal studies are needed to determine whether high‐γ trajectories can serve as a prognostic biomarker of motor‐complication vulnerability.

### Clinical and Translational Implications

The identification of opposing, stage‐dependent trajectories in the α‐ and β‐band networks provides a functional framework for tracking complementary phenotypic axes in PD. α‐Network integrity appears closely linked to cognition and postural‐gait dysfunction, whereas β‐network hypersynchronization aligns with bradykinesia severity. Their presence at the earliest disease stage and progressive evolution across stages underscore the potential as early diagnostic markers and longitudinal biomarkers of disease progression. Findings in the high‐γ band add further translational relevance: as stronger high‐γ connectivity may confer resilience against motor complications, non‐invasive neuromodulation strategies aimed at enhancing or preserving high‐γ activity within this compensatory network may be tested to prevent or delay such complications.^39^

The predictive analyses further strengthen the translational value of frequency‐specific EEG‐based connectivity measures. Compared with single‐band approaches, the multiband classifier achieved superior discriminative performance, both in differentiating PD stages within the patient cohort and, most notably, in distinguishing ePD patients from HCs. This improved classification performance likely reflects the integration of biologically distinct network signatures, while mitigating the impact of noise and interindividual variability inherent in band‐specific neurophysiological markers. Classification performance for ePD versus HCs was near‐perfect in the primary whole‐cohort analysis and was confirmed in the complementary site‐based external validation. This is particularly relevant, as early PD is the stage at which objective, scalable, relatively inexpensive, and non‐invasive biomarkers have the greatest diagnostic utility. Accordingly, if confirmed in larger and multicenter cohorts, multiband EEG‐connectivity profiling may complement clinical, imaging, and fluid biomarkers for PD diagnosis.

Importantly, the robustness of these findings was supported by the analytical design. The primary classification analyses were conducted using a cross‐validation framework in the whole cohort, allowing all available data to contribute to model estimation. To address possible concerns regarding overfitting and site‐specific effects, we additionally performed a site‐based external validation analysis in which models were trained and optimized in the Padua cohort and subsequently tested in the independent Rome cohort. The preservation of high discriminative performance in this setting supports the generalizability of the identified EEG connectivity signatures.

Although ePD and aPD were identified with good‐to‐excellent accuracy, classification performance was consistently lower for intermediate‐stage patients, likely reflecting the intrinsic biological and clinical heterogeneity of this stage. Our results support conceptualizing PD progression as a dynamic continuum rather than as discrete stages, with intermediate patients occupying a transitional space characterized by overlapping network features.^6^ Reduced classification separability may itself represent a meaningful signal of network instability, reflecting the coexistence of compensatory and maladaptive mechanisms rather than a clearly demarcated disease phase.

Another clinically relevant implication arises from the ability of EEG‐derived networks to identify advanced disease stages. Current clinical criteria for advanced PD are designed to maximize sensitivity in identifying patients who may benefit from advanced therapies. However, these criteria are symptom‐based and are limited by reduced specificity, interindividual variability, and contextual factors such as treatment strategies.^18^ The high‐γ connectivity loss observed in our study provides a candidate physiological marker of compensatory failure, potentially capturing a biologically grounded transition point associated with exhaustion of adaptive cortical mechanisms. Integrating such physiological markers with clinical criteria could enable more objective identification of patients most likely to benefit from advanced therapies.

Finally, the predictive value of frequency‐specific networks for clinically relevant disease milestones highlights the importance of a pathophysiologically grounded, band‐selective approach. Preserved α‐band network integrity was associated with a high NPV for cognitive impairment and severe axial symptoms, suggesting that stronger α connectivity identifies patients less likely to develop these disabling features. Similarly, high‐γ network integrity predicted lower motor complications, suggesting a protective role against the fluctuations and dyskinesia. Overall, although multiband integration is advantageous for diagnostic discrimination and disease stratification, the prediction of specific clinical milestones is more effectively achieved by targeting the oscillatory network most closely linked to the underlying symptom domain.

### Limitations and Strengths

This study has some limitations. First, the cross‐sectional design precludes causal inferences about within‐subject trajectories. In addition, because groups were age‐matched, advanced‐stage patients necessarily had longer disease duration and younger age at onset than early‐stage patients, potentially enriching the aPD group with individuals characterized by distinct genetic or biological disease substrates. Longitudinal studies are, therefore, needed to confirm individual progression patterns. We did not include biological markers to directly link network alterations to neurotransmitter‐specific dysfunctions. Moreover, EEG assessment of high‐γ‐band connectivity may be affected by cranial or facial muscle activity, particularly in awake participants. Although we applied several methodological steps to minimize this potential confound (including artifact rejection, ICA‐based correction, source‐space reconstruction, and the use of wPLI to reduce the influence of 0‐lag volume conduction), residual muscle contamination cannot be entirely excluded. Finally, classification of the intermediate stage remains partly arbitrary, reflecting the lack of validated criteria defining a distinct mid‐stage PD phenotype.

Despite these limitations, the study has several strengths. We analyzed a large, well‐characterized cohort spanning multiple disease stages, enabling characterization of frequency‐specific network trajectories across disease severity. FC was assessed using source‐level HD‐EEG with a fully standardized preprocessing and connectivity pipeline, enhancing reproducibility. The integration of NBS with ML enabled the identification of stage‐dependent network signatures while reducing reliance on arbitrary thresholds. Although EEG recordings were standardized in the OFF‐medication state, thereby limiting acute treatment effects, and no significant associations were observed between LEDD and the main connectivity measures, more subtle confounding related to chronic dopaminergic treatment burden or drug class‐specific effects cannot be entirely excluded. A further strength is that the present patient cohort does not overlap with those included in our previous studies, therefore, providing independent confirmation of α‐ and β‐band abnormalities in early PD. Finally, frequency‐resolved analyses combined with a multiband classifier captured complementary information across oscillatory systems. Overall, these strengths support the robustness, reproducibility, and translational potential of network‐based, frequency‐specific connectivity markers in PD.

## Conclusions

HD‐EEG–based functional connectivity reveals biologically meaningful, stage‐dependent network alterations in PD, with frequency‐specific patterns mapping onto distinct pathophysiological mechanisms and clinically relevant symptom domains. Progressive α‐band hypoconnectivity tracks cognitive decline and PIGD, whereas β‐band hyperconnectivity parallels the bradykinesia severity. In contrast, high‐γ dynamics are consistent with a compensatory cortical network mitigating motor complications, which declines as the disease progresses. Beyond the diagnostic value of individual networks, integrating multiband connectivity in a single model improved discriminatory performance, indicating that combined frequency‐specific signatures provide a more comprehensive and clinically actionable characterization of disease stage. Together, these findings endorse HD‐EEG connectivity as a non‐invasive, scalable, and pathophysiologically grounded biomarker candidate for PD diagnosis, staging, patient stratification, and progression monitoring.

## Author Contributions

M.Co., A.G., A.S., M.P., T.S., D.C., M.Cor., and A.A. contributed to the conception and design of the study; M.Co., V.D., L.L., L.L.G., D.M., V.F., C.S., S.P., F.D.G., M.P., T.S., A.S., and A.G. contributed to the acquisition and analysis of data; M.Co., D.M., A.G., A.S., and M.Cor. contributed to drafting the text or preparing the figures.

## Potential Conflicts of Interest

Nothing to report.

## Supporting information


**Figure S1.** Receiver operating characteristic (ROC) curves for site‐based external classification performance based on NBS‐derived EEG functional networks. Panels A–D show ROC curves for group‐level classification tasks, including differentiation between ePD and HC (A), and one‐vs‐rest classification of ePD (B), iPD (C), and aPD (D). For each panel, classification performance is reported for polynomial‐kernel SVM models, trained and optimized in the Padua cohort (n = 140) and externally validated in the independent Rome cohort (n = 57), on frequency‐specific NBS networks in the *α* (red), *β* (blue), and high‐*γ* (yellow) bands, as well as for a multiband model integrating all frequency‐specific predictors (magenta).
**Table S1.** Site‐based external validation performance of frequency‐specific and multiband NBS‐derived networks in differentiating ePD patients from HCs. The table reports performance metrics from polynomial‐kernel SVM models trained and optimized in the Padua cohort (n = 80) and externally validated in the independent Rome cohort (n = 37). Models were trained using *α*‐, *β*‐, and high‐*γ*‐band NBS‐derived networks, as well as a multiband model integrating all frequency‐specific predictors.
**Table S2.** Site‐based external validation performance metrics (one‐vs‐rest) for each PD group (ePD, iPD, aPD) across frequency‐specific NBS networks and the combined multiband model. The table reports performance metrics for each PD group (ePD, iPD, aPD) using polynomial‐kernel SVM models trained and optimized in the Padua cohort (n = 100) and externally validated in the independent Rome cohort (n = 40). Models were trained on *α*‐, *β*‐, and high‐*γ*‐band NBS‐derived networks, as well as on a multiband model integrating all frequency‐specific predictors.

## Data Availability

The raw data supporting the conclusions of this article will be made available by the corresponding author on reasonable request for purposes of replication and re‐use, subject to approval by the relevant ethics committees and applicable data‐protection regulations.
