# Supplementary material for: Frequency‐ and Network‐Specific Changes in Functional Connectivity Reflect Pathophysiological Mechanisms across Parkinson's Disease Stages
Source: Ann Neurol. 2026 May 27;100(2):319–33. doi: 10.1002/ana.78262 (PMC13387975; doi:10.1002/ana.78262)
Supplement: Supplementary file 1 — Figure S1. Receiver operating characteristic (ROC) curves for site‐based external classification performance based on NBS‐derived EEG functional networks. Panels A–D show ROC curves for group‐level classification tasks, including differentiation between ePD and HC (A), and one‐vs‐rest classification of ePD (B), iPD (C), and aPD (D). For each panel, classification performance is reported for polynomial‐kernel SVM models, trained and optimized in the Padua cohort (n = 140) and externally validated in the independent Rome cohort (n = 57), on frequency‐specific NBS networks in the α (red), β (blue), and high‐γ (yellow) bands, as well as for a multiband model integrating all frequency‐specific predictors (magenta). Table S1. Site‐based external validation performance of frequency‐specific and multiband NBS‐derived networks in differentiating ePD patients from HCs. The table reports performance metrics from polynomial‐kernel SVM models trained and optimized in the Padua cohort (n = 80) and externally validated in the independent Rome cohort (n = 37). Models were trained using α‐, β‐, and high‐γ‐band NBS‐derived networks, as well as a multiband model integrating all frequency‐specific predictors. Table S2. Site‐based external validation performance metrics (one‐vs‐rest) for each PD group (ePD, iPD, aPD) across frequency‐specific NBS networks and the combined multiband model. The table reports performance metrics for each PD group (ePD, iPD, aPD) using polynomial‐kernel SVM models trained and optimized in the Padua cohort (n = 100) and externally validated in the independent Rome cohort (n = 40). Models were trained on α‐, β‐, and high‐γ‐band NBS‐derived networks, as well as on a multiband model integrating all frequency‐specific predictors. [file ANA-100-319-s001.docx]

**Supplementary Materials**

**Cortical source localization**

Each participant underwent a structural MRI, with individual T1-weighted MPRAGE scans. The headmodel and the cortex surface were computed for each subject using Freesurfer software^1^. Then, the personal MRI sequence and EEG data were co-registered using corresponding anatomical landmarks. The forward model was solved using the Boundary Element Method (BEM)^2^, and the inverse solution was computed using weighted minimum-norm estimation (wMNE)^3^. Reconstructed sources were parcellated into 68 cortical regions according to the Desikan–Killiany atlas ^4^.

**Functional Connectivity (FC) analysis**

FC was estimated in source space using the weighted phase lag index (wPLI), which mitigates the influence of volume conduction, noise, and small-sample bias ^5^. Phase information was extracted with the Hilbert transform in the θ (4–8 Hz), α (8–13 Hz), β (13–30 Hz), low-γ (30–50 Hz), and high-γ (50–100 Hz) bands. Notably, γ activity was analyzed separately in low- and high-γ bands, as converging evidence suggests that these frequency bands are associated with distinct functional roles. Low-γ activity is preferentially associated with associative processing, while high-γ activity is more closely related to motor execution ^6,7^. Following Welch’s method, dynamic FC matrices were computed for each pair of regions using 1-second windows with 50% overlap, and then averaged across windows to obtain static FC matrices ^8^. All analyses were performed using the Brainstorm toolbox supplemented with custom MATLAB R2025b scripts.

**Network-Based Statistic (NBS) approach**

NBS is a cluster-based statistical method used in several previous studies ^9–11^, which provides greater statistical power than standard univariate tests and traditional correction methods ^12^.

First, in the NBS algorithm, the difference in each-band FC between ePD, iPD, aPD, and HC of each edge was analyzed using ANCOVA, using age and sex as covariates. Next, we computed the network size whose edges had greater weights than the defined F threshold. We then performed a permutation test, randomly assigning all subjects to one of the four groups, maintaining each group size N-1 times, and computed the maximum sizes of networks whose edges had weights greater than the threshold, resulting in an empirical null distribution of maximum sizes. The number of permutations used in the present study was 5000. Then, we assigned the p-value of the network with a fraction of the occurrences whose sizes were larger than the size of the network of the original assignment. Moreover, we estimated the effect size of the NBS network by averaging the effect sizes across all network edges, as previously described ^9^. The effect size for the ANCOVA test of each edge was assessed using partial eta-squared (ηp²). The conventional magnitude thresholds are reported above.

**Machine Learning–Based Network Selection**

Although control of the family-wise error rate (FWER) is guaranteed irrespective of the NBS threshold, choosing the NBS threshold represents an arbitrary parameter. To address this issue, we proposed an approach that combines NBS with ML protocols^13,14^. Among the candidate methods, support vector machines (SVMs) consistently showed superior performance, and a detailed kernel comparison (linear, polynomial, and Gaussian radial function) revealed that the polynomial kernel provided the best predictive accuracy in our dataset.

Specifically, we employed a multiclass SVM classifier that included all diagnostic groups (HC, ePD, iPD, aPD). Model performance was evaluated using a nested cross-validation scheme: leave-one-out cross-validation (LOOCV) as the outer loop to obtain unbiased performance estimates, and an inner Bayesian optimization loop to automatically tune hyperparameters. This approach maximized generalization accuracy while minimizing the risk of overfitting, ensuring a robust and reproducible evaluation of the discriminative value of NBS-derived connectivity features.

To identify the optimal NBS-derived network, we examined a continuum of primary thresholds (from F = 2.0 in increments of 0.1) and computed, for each threshold, the corresponding node and link counts along with their p-values. As expected, several adjacent thresholds yielded statistically significant components (p < 0.05). For each significant network in this continuum, we applied the multiclass SVM classification procedure described above, including nested LOOCV and Bayesian hyperparameter optimization, to evaluate its predictive performance. In this way, classification accuracy served as an empirical criterion for selecting among competing NBS solutions. The network that achieved the highest cross-validated accuracy was retained as the optimal NBS network^9,13,14^.

**References**

1. Fischl, B. FreeSurfer. *NeuroImage* vol. 62 Preprint at https://doi.org/10.1016/j.neuroimage.2012.01.021 (2012).

2. Jatoi, M. A. *et al.* BEM based solution of forward problem for brain source estimation. *IEEE 2015 International Conference on Signal and Image Processing Applications, ICSIPA 2015 - Proceedings* 180–185 (2016) doi:10.1109/ICSIPA.2015.7412186.

3. Grech, R. *et al.* Review on solving the inverse problem in EEG source analysis. *J. Neuroeng. Rehabil.* **5**, 1–33 (2008).

4. Desikan, R. S. *et al.* An automated labeling system for subdividing the human cerebral cortex on MRI scans into gyral based regions of interest. *Neuroimage* **31**, (2006).

5. Hardmeier, M. *et al.* Reproducibility of functional connectivity and graph measures based on the phase lag index (PLI) and weighted phase lag index (wPLI) derived from high resolution EEG. *PLoS One* **9**, (2014).

6. Ball, T. *et al.* Movement related activity in the high gamma range of the human EEG. *Neuroimage* **41**, (2008).

7. Parciauskaite, V. *et al.* Individual resonant frequencies at low-gamma range and cognitive processing speed. *J. Pers. Med.* **11**, (2021).

8. Jwo, D. J., Chang, W. Y. & Wu, I. H. Windowing Techniques, the Welch Method for Improvement of Power Spectrum Estimation. *Computers, Materials and Continua* **67**, (2021).

9. Conti, M. *et al.* Cortical Functional Connectivity Changes in the Body‐First and Brain‐First Subtypes of Parkinson’s Disease. *Movement Disorders* **40**, 254–265 (2025).

10. Conti, M. *et al.* Band‐Specific Altered Cortical Connectivity in Early Parkinson’s Disease and its Clinical Correlates. *Movement Disorders* **38**, 2197–2208 (2023).

11. Yassine, S. *et al.* Identification of Parkinson’s Disease Subtypes from Resting-State Electroencephalography. *Movement Disorders* https://doi.org/10.1002/mds.29451 (2023) doi:10.1002/mds.29451.

12. Serin, E., Zalesky, A., Matory, A., Walter, H. & Kruschwitz, J. D. NBS-Predict: A prediction-based extension of the network-based statistic. *Neuroimage* **244**, (2021).

13. Conti, M. *et al.* Increased blood-brain barrier permeability is associated with dysfunctional α band connectivity in early-stage Parkinson’s disease. *J. Neural Transm.* https://doi.org/10.1007/s00702-025-03002-1 (2025) doi:10.1007/s00702-025-03002-1.

14. Conti, M. *et al.* Clinical, Biological, and Functional Connectivity Profile of Patients With De Novo Parkinson Disease Who Are *APOE* ε4 Carriers. *Neurology* **106**, (2026).

**
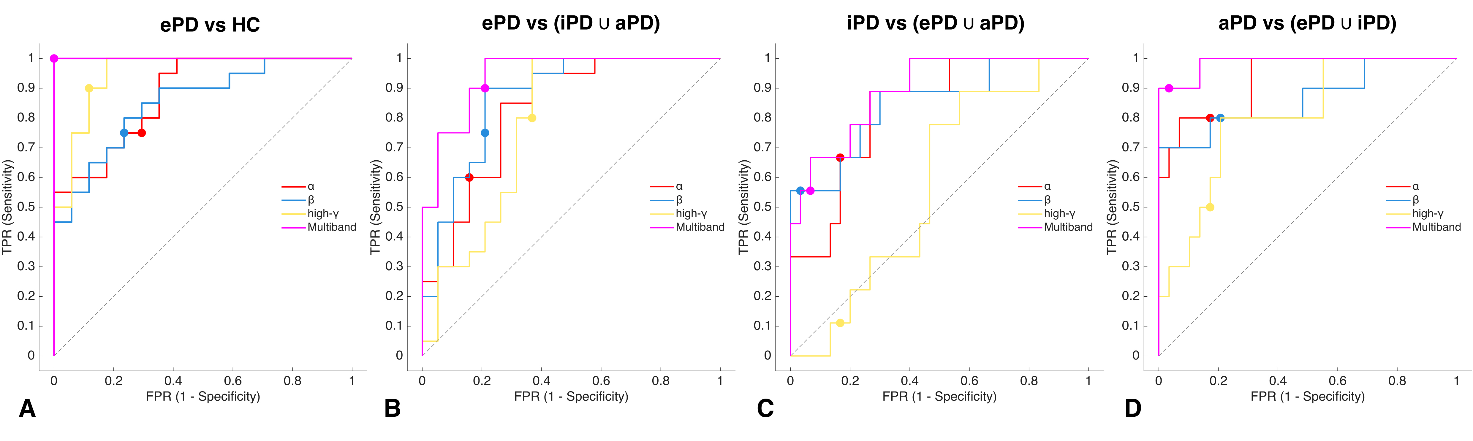
**

**Figure S1. Receiver operating characteristic (ROC) curves for site-based external classification performance based on NBS-derived EEG functional networks.** Panels A–D show ROC curves for group-level classification tasks, including differentiation between ePD and HC (A), and one-vs-rest classification of ePD (B), iPD (C), and aPD (D). For each panel, classification performance is reported for polynomial-kernel SVM models, trained and optimized in the Padua cohort (n=140) and externally validated in the independent Rome cohort (n=57), on frequency-specific NBS networks in the α (red), β (blue), and high-γ (yellow) bands, as well as for a multiband model integrating all frequency-specific predictors (magenta).

**Table S1. Site-based external validation performance of frequency-specific and multiband NBS-derived networks in differentiating ePD patients from HCs.** The table reports performance metrics from polynomial-kernel SVM models trained and optimized in the Padua cohort (n=80) and externally validated in the independent Rome cohort (n=37). Models were trained using α-, β-, and high-γ-band NBS-derived networks, as well as a multiband model integrating all frequency-specific predictors.

| **Metric** | **ePD vs HC** | | | |
| --- | --- | --- | --- | --- |
|  | **α** | **β** | **h-γ** | **Multi** |
| AUC | **0.88** | **0.85** | **0.95** | **1.00** |
| Sensitivity (TPR) | 0.75 | 0.75 | **0.90** | **1.00** |
| Specificity (TNR) | 0.71 | 0.76 | **0.88** | **1.00** |
| FNR (Type II error) | 0.25 | 0.25 | 0.10 | 0.00 |
| FPR (Type I error) | 0.29 | 0.24 | 0.12 | 0.00 |
| PPV | 0.75 | 0.79 | **0.90** | **1.00** |
| FDR | 0.25 | 0.21 | 0.10 | 0.00 |
| NPV | 0.71 | 0.72 | **0.88** | **1.00** |
| FOR | 0.29 | 0.28 | 0.12 | 0.00 |
| Accuracy | 0.73 | 0.76 | **0.89** | **1.00** |

AUC = Area Under the Curve; TPR = True Positive Rate (or Sensitivity); FNR = False Negative Rate (or Type II error); TNR = True Negative Rate (or Specificity); FPR = False Positive Rate (or Type I error); PPV = Positive Predictive Value; FDR = False Discovery Rate; NPV = Negative Predictive Value; FOR = False Omission Rate; Accuracy = Overall Correct Classification Rate.

**Table S2. Site-based external validation performance metrics (one-vs-rest) for each PD group (ePD, iPD, aPD) across frequency-specific NBS networks and the combined multiband model.** The table reports performance metrics for each PD group (ePD, iPD, aPD) using polynomial-kernel SVM models trained and optimized in the Padua cohort (n=100) and externally validated in the independent Rome cohort (n=40). Models were trained on α-, β-, and high-γ-band NBS-derived networks, as well as on a multiband model integrating all frequency-specific predictors.

| **Metric** | **ePD vs (iPD ∪ aPD)** | | | | **iPD vs (ePD ∪ aPD)** | | | | **aPD vs (ePD ∪ iPD)** | | | |
| --- | --- | --- | --- | --- | --- | --- | --- | --- | --- | --- | --- | --- |
|  | **α** | **β** | **h-γ** | **Multi** | **α** | **β** | **h-γ** | **Multi** | **α** | **β** | **h-γ** | **Multi** |
| AUC | **0.83** | **0.87** | 0.78 | **0.94** | **0.83** | **0.85** | 0.57 | **0.89** | **0.93** | **0.87** | **0.80** | **0.99** |
| Sensitivity (TPR) | 0.60 | 0.75 | **0.80** | **0.90** | 0.67 | 0.56 | 0.11 | 0.56 | **0.80** | **0.80** | 0.50 | **0.90** |
| Specificity (TNR) | **0.84** | 0.79 | 0.63 | 0.79 | **0.83** | **0.97** | **0.83** | **0.93** | **0.83** | 0.79 | **0.83** | **0.97** |
| FNR (Type II error) | 0.40 | 0.25 | 0.20 | 0.10 | 0.33 | 0.44 | 0.89 | 0.44 | 0.20 | 0.20 | 0.50 | 0.10 |
| FPR (Type I error) | 0.16 | 0.21 | 0.37 | 0.21 | 0.17 | 0.03 | 0.17 | 0.07 | 0.17 | 0.21 | 0.17 | 0.03 |
| PPV | **0.80** | 0.79 | 0.70 | **0.82** | 0.55 | **0.83** | 0.17 | 0.71 | 0.62 | 0.57 | 0.50 | **0.90** |
| FDR | 0.20 | 0.21 | 0.30 | 0.18 | 0.45 | 0.17 | **0.83** | 0.29 | 0.38 | 0.43 | 0.50 | 0.10 |
| NPV | 0.67 | 0.75 | 0.75 | **0.88** | **0.89** | **0.88** | 0.76 | **0.88** | **0.92** | **0.92** | **0.83** | **0.97** |
| FOR | 0.33 | 0.25 | 0.25 | 0.12 | 0.11 | 0.12 | 0.24 | 0.13 | 0.08 | 0.08 | 0.17 | 0.03 |
| Accuracy | 0.72 | 0.77 | 0.72 | **0.85** | 0.79 | **0.87** | 0.67 | **0.85** | **0.82** | 0.79 | 0.74 | **0.95** |

AUC = Area Under the Curve; TPR = True Positive Rate (or Sensitivity); FNR = False Negative Rate (or Type II error); TNR = True Negative Rate (or Specificity); FPR = False Positive Rate (or Type I error); PPV = Positive Predictive Value; FDR = False Discovery Rate; NPV = Negative Predictive Value; FOR = False Omission Rate; Accuracy = Overall Correct Classification Rate.
